# Can International Classification of Functioning, Disability and Health (ICF) Be Used for Prediction of Work Capacity and Employment Status in Multiple Sclerosis?

**DOI:** 10.3390/jcm13144195

**Published:** 2024-07-18

**Authors:** Daiva Valadkevičienė, Dalius Jatužis, Irena Žukauskaitė, Indre Bileviciute-Ljungar

**Affiliations:** 1 Clinic of Neurology and Neurosurgery, Institute of Clinical Medicine, Faculty of Medicine, Vilnius University, LT-03101 Vilnius, Lithuania; 2 The Agency for Protection of the Rights of Persons with Disabilities at the Ministry of Social Security and Labour of the Republic of Lithuania, LT-03223 Vilnius, Lithuania; 3 Institute of Psychology, Faculty of Philosophy, Vilnius University, LT-01513 Vilnius, Lithuania; 4 Department of Clinical Science, Karolinska Institutet, SE-18288 Stockholm, Sweden; 5 Multidisciplinary Pain Clinic, Capio St. Göran Hospital, SE-11291 Stockholm, Sweden

**Keywords:** multiple sclerosis, employment, work capacity evaluation, disability evaluation, international classification of functioning, disability and health, activities of daily living, humans

## Abstract

**Background:** Multiple sclerosis (MS) affects many body functions and activities, including work capacity and ability to work. An evaluation of work-related parameters is important to understand the barriers to maintaining the job. The aim of this study was to evaluate if a Comprehensive International Classification of Functioning, Disability and Health (ICF) core set for MS can be used to predict work capacity and employment status. **Methods:** The cohort included 151 participants with MS (99 female/52 male, mean age 49 years) referred for a work capacity evaluation. **Results:** 71 (47.0%) were employed and a major part (131, 86.7%) had a work capacity between 20 and 40% with no difference between those who were employed and those who were unemployed. The analysis revealed that age and the following categories explained 68.8% of the work capacity: b770 Gait pattern functions; b730 Muscle power functions; b134 Sleep functions; d845 Acquiring, keeping and terminating a job; and b620 Urination functions. The following categories in 79.5% predicted ability to work: b164 Higher-level cognitive functions; d510 Washing oneself; d630; Preparing meals; and d870 Economic self-sufficiency. **Conclusions:** Here, we show that different functions/activities predicted work capacity in comparison with employment status in MS. Therefore, ICF should be implemented when assessing work ability.

## 1. Introduction

Multiple sclerosis (MS) is a chronic autoimmune disease that causes lesions in the white matter of the brain and spinal cord due to immune cell infiltration and demyelination [1]. Dysregulation of the immune system is supposed to lead to the progression of MS [2]. Recent pharmacological treatment strategies are directed to break the disease by immune modulation/suppression or to promote remyelination/neuroprotection [3]. MS also affects cortical and deep grey matter and results in axonal damage to the brain and spinal cord [4]. This creates disintegration of central nervous system functions [5]. This disintegration of neuronal networks is another sign of the progression of MS leading to reduced brain volume and atrophy [6]. Therefore, damage to the central nervous system may affect important body functions for daily life functioning. The prevalence of MS-related fatigue is suggested to vary between 37 and 78% according to the systemic literature review, which included studies between 2010 and 2020 [7]. The study also identified significant associations between MS-related fatigue and lower quality of life, as well as employment status, work capacity and sick leave. Lesions in the white matter of brain structures with thalamic atrophy are suggested to play a major role in cognitive impairments in early MS [8]. For example, demyelination, microstructural changes, neuronal damage and disconnection of the hippocampus from several brain networks might explain fatigue, and affective and cognitive impairments in MS [9,10]. In a self-reported Danish survey, fatigue, pain and sleep disturbances were associated with lower quality of life in 2009 patients with MS [11]. Altogether, this indicates the need for broader assessments of body functions and participation when evaluating individuals with MS since multiple and varying types of damage of the nervous system in MS affect many body functions resulting in a variety of symptoms [12] and disability levels [13]. Moreover, MS usually starts in younger adults (between 20 and 40 years old) [14], which has a huge impact on work capacity and maintaining a job. Apart from structural lesions in the central nervous system, many other factors have been shown to affect work capacity in individuals with MS. Reports show that age, fatigue, disability, comorbidities and mental health [15,16,17] are important for work capacity in MS, while other studies show that cognitive impairments also play an important role [18], or even predict work disability [19,20]. Other factors, which influenced employment status at 3 years follow-up, were increased depression, higher impact of fatigue, more cognitive complaints and less workplace support [21].

Multiple components of this neurodegenerative disorder of the central nervous system should be assessed with a biopsychosocial model using the WHO’s International Classification of Functioning and Disability (ICF). The ICF was officially endorsed by all 191 WHO Member States at the 54th World Health Assembly on 22 May 2001 (resolution WHO 54.21) [22] and can be used for clinical practice and research. It includes evaluation of functioning, activity, participation and environmental factors. For many years, the ICF core set has been suggested to be used for MS patients as a tool to assess impaired body function, reduced activity and restricted participation [23,24]. A Comprehensive ICF core set for MS was validated in a three-round Delphi technique [25], though clinical use of this tool is still very limited. However, the use of ICF has been recently suggested to be an effective method to determine relevant outcomes for clinical trials in MS rehabilitation interventions [26].

Since ICF is a tool to evaluate multiple functions and activity/participation categories and environmental factors, it could be useful for identifying factors related to work capacity prediction. Reports show the implementation of ICF in a work ability assessment [27] or in communication with job centres when MS people were involved in the return-to-work process [28]. However, studies regarding the application of ICF in predicting work ability in particular disorders, including MS, are absent. Studies on the environmental factors for MS are few [29], and personal factors could not be classified by ICF but, in general, have been shown to stimulate return to work [30]. 

Recently, we have investigated whether categories included in the Brief ICF core set for MS (20 categories) predicted the worsening of MS one year later and found that category b164 (higher-level cognitive functions) predicted MS progression [31]. However, the functions and activities with the highest levels of impairment were identified in relation to ICF categories related to “energy and drive functions”, “muscle and power functions”, and “moving around” [31], which did not predict progression one year later. When studying a Comprehensive ICF core set for MS (138 categories), we found that activities related to “walking” (d450), “moving around” (d455), “moving around using equipment” (d465), “handling stress” (d240), and “recreation and leisure” (d920) were the categories showing the highest levels of impairments [32]. Our previous findings suggest that the activity and participation evaluated using an ICF core set could be predictive of work capacity in MS.

The aim of this study was to evaluate if a Comprehensive ICF core set for MS can be used to predict work capacity and employment status. Therefore, a secondary analysis that relied on multiple and logistic regression models and included sociodemographic, clinical and ICF-related data was applied to the originally published results [32] and analysed in relation to work capacity and employment status. Our hypothesis was that the evaluation of participants´ functioning and disability as well as environmental factors will capture the main predictors of work capacity and employment status. In other words, this study aimed to analyse the complex picture of MS disability from the ICF point of view regarding work-related factors. 

## 2. Materials and Methods

This descriptive cross-sectional single-centre study included 151 participants with MS referred to the Agency for Protection of the Rights of Persons with Disabilities (APRPD, previously the Disability and Working Capacity Assessment Office, DWCAO, under the Ministry of Social Security and Labour of the Republic of Lithuania). Since the purpose of the study was a secondary analysis of previously reported data, we present only those methods which were used to achieve the aims of the present study. Therefore, a complete description regarding recruitment procedure, study design and implementation of the ICF Comprehensive Core set for MS has already been reported [32]. The summary of recruitment is (repeatedly) presented in Supplementary Figure S1.

### 2.1. Sociodemographic Data, Clinical Data and Evaluation of ICF Categories

As reported previously, sociodemographic data (age, sex and work capacity data) and clinical data (type of MS, disease duration, comorbidities, medication and aids to improve function and disability) were collected by the main investigator (DV) from journal records at APRPD. The data on employment status were collected from journal recordings at APRPD and confirmed following the interview. Comprehensive ICF data for the MS participants were collected by the main investigator (DV) during telephone interviews due to restrictions on face-to-face interaction during the COVID-19 pandemic. Additional information was extracted from the journal records at APRPD regarding the Expanded Disability Status Scale (EDSS) [33] and categories related to “defecation functions” (b525), “urination functions” (b620), “muscle power functions” (b730), “muscle tone functions” (b735), “muscle endurance functions” (b740), “motor reflex functions” (b750), “control of voluntary movement functions” (b760), “gait pattern functions” (b770), “structure of brain” (s110) and “spinal cord and related structures” (s120). Due to a lack of available information and restrictions to assess participants during physical appointments, 125 of 138 ICF categories were evaluated. For the ICF in general, the personal factors could not be classified and, therefore, were not assessed in this study.

### 2.2. Assessment of Work Capacity at APRPD

The work capacity level was set using 5% intervals in accordance with legal regulations on the process of setting the work capacity level. A range of 0–25% work capacity indicates that an individual cannot work or only has the ability to work in an environment that has been adapted to the specific disability. A range of 30–55% indicates that an individual has a reduced level of work capacity, meaning that an individual is able to work in an environment with adaptations or no adaptations. A range of 60–100% indicates that a person has full work capacity. It is noteworthy that differences in the work capacity range are associated with different monetary allowances for the patients. 

According to legal regulations, the validity of a work capacity level assessment may last for one or two years or, if there are no indications of a capacity for improvement, can be permanent (until the end of life). Nevertheless, patients with a permanent work capacity assessment may seek out an additional assessment at the APRPD if they believe their condition has changed. 

In terms of the periodicity of assessment, the primary evaluation is conducted during the first visit and when the decision on the level of impairment is made. A repeat evaluation of work capacity is conducted at the end of the validity period of the primary evaluation or after any changes in an individual’s health condition, changes in the original reasons for the work capacity level, or if a person or the funding institution does not accept the results of the APRPD’s evaluation. 

During the study period (2022), the retirement age was 64 years and 4 months for men and 63 years and 8 months for women. 

This study was conducted according to an agreement between Vilnius University and APRPD (DWCAO) signed on 17 November 2021 [(No. (5.74) SU-2990)]. This study was approved by the Lithuanian Bioethics Committee (No. 2021/10–1387-855), and each participant signed an informed consent and an agreement regarding personal data usage.

### 2.3. Statistical Analyses

Descriptive statistics were used to describe the study population and report frequencies of the most relevant ICF categories in participants with MS. Multiple regression analyses (linear model with stepwise selection) were performed to identify a set of ICF categories that best differentiate between different levels of work capacity. ICF categories to be entered in regression analyses were selected as follows: 

First, only ICF categories representing a problem for over 10% of patients were analysed. 

Second, ICF categories obtaining a coefficient of Spearman correlation rs ≥ 0.25 (*p* > 0.05) in relation to work capacity were selected for further analysis. 

Age, gender and time from diagnosis were included in the regression analyses as forced-in variables. As the variance inflation factor (VIF) was below four in all models, we considered collinearity to be no problem in our analyses.

Logistic regression analysis (forward Wald) was conducted to predict the employment status of participants with MS. Hosmer and Lemeshow Test, correct classification rate and Nagelkerke regression coefficient (as normalised Cox and Snell pseudo-R2) were selected as model fit measures.

Data analyses were performed with SPSS (version 22).

## 3. Results

### 3.1. Sociodemographic and Clinical Data

Data from 151 participants with MS were analysed [mean age = 49.3 years; *n* = 52 male (34.4%)]. Participants’ sociodemographic and health-related characteristics are presented in Table 1, which shows that participants had an average EDSS score of 4.6. The majority of participants had relapsing-remitting multiple sclerosis (RRMS) (80%) and were treated with disease-modifying treatment (over 80%). Almost 75% of participants did not have any comorbidities. There was a significant difference in age, time of symptoms, time from diagnosis, EDSS score and educational status between participants who worked and those who did not. Participants receiving disease-modifying treatment were more likely to be employed than those who were not receiving treatment.

**Figure 1:**
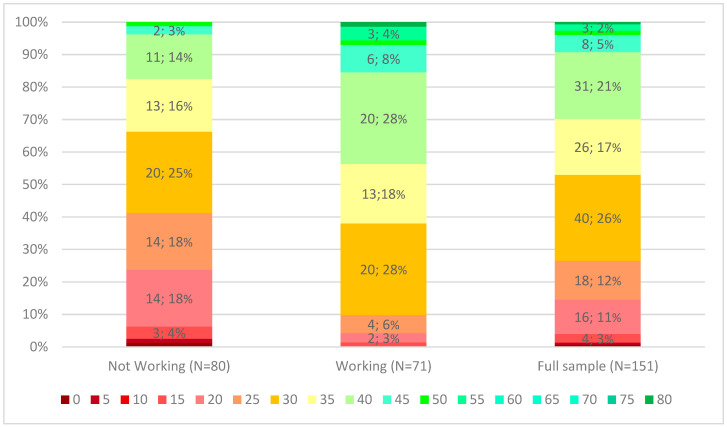
Distribution of different work capacity levels in study groups.

The results show that a major part of the participants (131; 86.7%) had a work capacity between 20 and 40% and 71 (47.0%) were employed. No difference was found in work capacity between those who were employed and those who were unemployed (Figure 1). 

### 3.2. Analysis of ICF Categories

Over 10% of participants had problems in 79 (63.2%) of the 125 ICF categories in the Comprehensive ICF core set for MS. These ICF categories are shown in Supplemental Table S1.

The 75 ICF categories (31 Body functions, 1 Body structures, 36 Activities and participation, and 7 Environmental factors) were considered for further analysis. Categories s120, d465 and d475 were excluded due to a small sample size and e590 was excluded due to an overlapping study object. The results show that 43 ICF categories (14 Body functions, 29 Activities and participation categories) attained a correlation coefficient of rs ≥ 0.25 (*p* < 0.05) for work capacity and were selected for further analysis (Table 2).

### 3.3. Multivariate Analyses for Work Capacity

The selected 43 ICF categories with a correlation coefficient of rs ≥ 0.25 (*p* < 0.05) for work capacity were entered into the stepwise regression models for predicting work capacity. Due to a high number of variables, only the Body functions category was entered into the analysis in the first step. With a regression coefficient of R^2^ = 0.648 [F (5) = 67.1, *p* < 0.001], only five variables were predictors of work capacity: b770 Gait pattern functions, b730 Muscle power functions, b134 Sleep functions, b620 Urination functions, and b164 Higher-level cognitive functions. The second regression analysis was only conducted for the categories Activities and Participation. With a regression coefficient R^2^ = 0.495 [F (3) = 48.0, *p* < 0.001], only three variables were predictors of work capacity: d460 Moving around in different locations; d640 Doing housework; and d845 Acquiring, keeping and terminating a job. Finally, all of the above variables, as well as sex, education, age of study participants, time from diagnosis, DMT and comorbidities, were entered into the final regression analysis. The results are presented in Table 3. The model fit is good [F (6) = 48.4, *p* < 0.001] with a regression coefficient of R^2^ = 0.668.

### 3.4. Multivariate Analyses for Active Employment

The final step of the analysis was to evaluate the predictors of employment status. The logistic regression analysis was conducted following the same steps as for multiple regression analysis. Firstly, only the Body functions categories were entered into the analysis. With Nagelkerke R^2^ = 0.275 and a Hosmer and Lemeshow Test = 0.301, only three variables were significant for prediction: b164 Higher-level cognitive functions, b760 Control of voluntary movement functions, and b770 Gait pattern functions. Secondly, only Activities and Participation categories were entered into the analysis, excluding d845 Acquiring, keeping and terminating a job and d850 Remunerative employment, due to overlap with the study object.

With Nagelkerke R^2^ = 0.574 and a Hosmer and Lemeshow Test = 0.240, only seven variables were significant for prediction of employment status: d330 Speaking, d455 Moving around, d510 Washing oneself, d630 Preparing meals, d720 Complex interpersonal interactions, d870 Economic self-sufficiency, and d910 Community life.

Finally, all of the above variables, as well as sex, education, age of study participants, time from diagnosis, DMT and comorbidities, were entered into the final logistic regression analysis. The results are presented in Table 4. The model fit is good (Hosmer and Lemeshow Test = 0.524) with a Nagelkerke regression coefficient of R^2^ = 0.677. The correct classification is 84.8%.

### 3.5. Summary of the Results

In conclusion, multiple analysis methods were applied to discover the most important ICF categories predicting the work capacity and employment status in participants with MS. Our results indicate that work capacity is mainly predicted by age and impairments of body functions (b-categories), while employment status is predicted mainly by education and ICF activities/participation categories (d-categories). 

## 4. Discussion 

### 4.1. Predictors of Work-Related Parameters in MS

The results of this study present a complex interaction between MS-related disability measured by the ICF Comprehensive Core set for MS and work-related parameters such as working capacity and employment status in a cohort referred to a work capacity evaluation agency in Lithuania. Almost half of the study population (47%) was employed. The average work capacity in the study cohort was between 20 and 40% with no difference between those who had a job and those who did not have a job. However, those who had jobs were younger, had a higher level of education, and had a shorter duration of MS symptoms. The analysis identified that age and the following five ICF categories were predictors of work capacity: b770 Gait pattern functions; b730 Muscle power functions; b134 Sleep functions; b620 Urination functions; and d845 Acquiring, keeping and terminating a job. 

Moreover, the results show that level of education and the following ICF categories were predictors of employment: b164 Higher-level cognitive functions, d330 Speaking, d455 Moving around, d510 Washing oneself, d630 Preparing meals, d870 Economic self-sufficiency, and d910 Community life. 

Other studies have identified similar factors contributing to work status. For example, an American cohort study with 8004 survey participants found that age, age at diagnosis, cognitive and hand function impairment, fatigue, higher disability levels and comorbidities, female sex, and PPMS were associated with not working [34]. Elderly MS patients have been reported to have a lower level of education and lower income in a Danish population of 8215 MS patients [35]. Clinical course (PPMS versus RRMS), age and number of years from MS diagnosis have been associated with higher sick leave and disability pension in a Swedish cohort of 5371 patients with MS [36]. We were not able to find any association between the type of MS and work capacity or employment status, which may be due to the smaller cohort size. Furthermore, evaluation of cognitive function with a more specific test (symbol digit modalities test) has been shown to be predictive of work capacity and has been suggested to be used as a routine measure for MS patients [19,20]. Recent studies indicate a role of psycho-emotional status, including behavioural coping strategies, in MS disability [17,37,38]. A recent review summarises the importance of linking MS symptomatology (for example, depressive symptoms) to ICF domains [39]. However, we were not able to find any association between ICF categories related to emotional status and work capacity or employment status as recently presented. The results of the present study analysed different functional and environmental ICF categories as predictors for work capacity and employment status. A recent systematic review identified job characteristics, work environment, social relationships at work, negative work events and lack of information as the most important barriers to work [37]. However, in the present study, the environmental categories, including work-related barriers or facilitators, were not identified as predictors. A previous review by Raggi et al. [29] reported few studies dealing with environmental/contextual factors regarding work-related problems in MS [29]. This indicates a need for more research in this area. 

### 4.2. The Use of ICF Core Sets in MS Research and Clinical Practice

However, few studies report the use of ICF in clinical practice and research. This may be due in part to the high number of categories (138) in the Comprehensive ICF core set for MS, which can make the assessment time-consuming [32,40]. When analysing the Comprehensive ICF core set for MS in 205 MS patients, Conrad et al. identified several categories not included in the Brief ICF core set as important for patients´ functioning and disability, including b620 urination functions, d230 carrying out daily routine, and d870 economic self-sufficiency [40]. In the present study, these categories have also been found to be important predictors of work capacity and the ability to keep a job. On the other hand, the categories in the Brief ICF core set for MS were not always associated with the participant´s functioning [40]. Therefore, research on the Brief ICF core set with only 20 categories should also be expanded to determine the most important categories reflecting functioning and disability, including work capacity and absenteeism in MS. Therefore, there is a need to improve the effectiveness of the implication of ICF regarding the number of necessary categories and the way of assessing the impairments. The introduction of Artificial Intelligence (AI) is challenging in rehabilitation medicine and disability research but could mean a considerable effectivisation in clinical practice and research when implementing ICF. 

After analysing data from participants with MS from APRPD, we recently proposed that several categories be added to the Brief ICF core set to cover the complexity of impaired categories. However, this should be confirmed by other studies. A previous report on the Comprehensive ICF for MS validation in 150 patients at a rehabilitation clinic found similar results in body function impairments, and reduced activities and participation, [31] with the exception of b280 sensation of pain [41]. Therefore, different cohorts can increase our knowledge of the most important ICF categories for MS and help update the Brief ICF core set. The latter study also showed that d850 remunerative employment; d870 economic self-sufficiency; and d845 acquiring, keeping and terminating a job were the most frequently reported restrictions in activities. Moreover, the ICF components correlated with EDSS, 6MWT (6 min walking test), and other scales, indicating that ICF is useful in the assessment of MS patients [41]. 

### 4.3. Limitations and Strengths of the Study

One of the most interesting findings of this study is that different ICF categories predicted work capacity and the ability to maintain a job. It is interesting to note that work capacity was mainly predicted by impairments in certain body functions that are more specific to MS (muscle/gait pattern functions and urination), while maintaining a job was predicted mainly by impairments in activities and participation important in daily life. 

Today, Lithuania provides a range of benefits to persons with disabilities from cash benefits to services, including medical and vocational rehabilitation, employment support, care allowance, support for housing, etc. [42]. Interestingly, labour market participation (employment) has no impact on eligibility for benefits, except for social assistance disability pension, which cannot be received while a person is working. Overall, this is a good feature of the disability system in Lithuania, as in many countries, people with disabilities must choose between disability pension and labour market participation [42]. This could explain our contradictory results where different ICF categories predicted work capacity and work ability. Telephone interviews were used due to pandemic restrictions, which is a clear limitation. Another limitation is the makeup of the study cohort, as only participants referred for work capacity assessment at APRPD under the Ministry of Social Security and Labour of the Republic of Lithuania were recruited. This together with the comparatively small study sample limits the generalizability of the results. Investigation of larger sample sizes from different countries and cohorts could result in more generalisable results promoting a common language of disability for patients, clinicians and stakeholders. 

The evaluation of ICF categories was performed by the same evaluator, which could be considered both a strength and a limitation. On the other hand, assessments of body functions, activity and participation, and environmental factors were performed using a strict study protocol that has recently been published by Elsevier [32]. Important work-related measures were assessed by an independent evaluator at APRPD and clinically relevant data (including some ICF categories) were collected from journal recordings, which increases the objectivity of collected data.

## 5. Conclusions

The results show that different ICF categories predicted work capacity in comparison to employment status in participants with MS. Using ICF assessments for functions and activities/participation might help clinicians predict work-related parameters and plan interventions, including tailored and occupational rehabilitation. Identifying impairments in ICF categories for work-related parameters is a new way to capture an increased risk for coming disability. However, more studies are needed to confirm these findings when identifying the most important ICF categories for use in the assessment of work capacity and actual employment status. Our research suggests that ICF might be useful when assessing prediction for both clinical and work-related parameters. Future results should concentrate on the implementation of ICF evaluation in clinical practice, for example, using commonly recognised protocols when assessing impairments in ICF categories. This will help to compare disability and environmental factors in different MS cohorts. In the next step, AI tools should be able to simplify and promote the clinical use of ICF when assessing and integrating both clinical, laboratory and functional parameters in MS patients. Our results show the importance of using a biopsychosocial ICF model to capture impairments in functions, limitations in activities and restrictions in participation in people with MS. 

## Figures and Tables

**Table 1:** Clinical and sociodemographic characteristics of full sample and groups of different employment status.

Variables		Full Sample (*n* = 151)	Working (*n* = 71)	Not Working (*n* = 80)	*p*
Age, years (mean ± SD)		49.3 ± 10.5	46.58 ± 11.00	51.66 ± 9.55	0.003
Time from symptoms, years (mean ± SD)		13.6 ± 9.1	11.34 ± 7.11	15.52 ± 10.18	0.004
Time from diagnosis, years (mean ± SD)		11.3 ± 8.0	9.01 ± 6.09	13.33 ± 8.95	0.001
EDSS score		4.6 ± 1.3	4.08 ± 1.05	5.12 ± 1.35	0.000
Sex	Male	52 (34.4%)	23 (32.4%)	29 (36.3%)	0.619
Education	Primary	7 (4.6%)	0 (0.0%)	7 (8.8%)	0.000
	Secondary	16 (10.6%)	7 (9.9%)	9 (11.3%)	
	Vocational	40 (26.5%)	8 (11.3%)	32 (40.0%)	
	College	30 (19.9%)	14 (19.7%)	16 (20.0%)	
	Higher	58 (38.4%)	42 (59.2%)	16 (20.0%)	
Type of disease	SPMS	19 (12.6%)	5 (7.0%)	14 (17.5%)	0.147
	PPMS	11 (7.3%)	5 (7.0%)	6 (7.5%)	
	RRMS	121 (80.1%)	61 (85.9%)	60 (75.0%)	
DMT	Moderate efficacy	66 (43.7%)	32 (45.1%)	34 (42.5%)	0.077
	High efficacy	57 (37.7%)	31 (43.7%)	26 (32.5%)	
	Untreated	28 (18.5%)	8 (11.3%)	20 (25.0%)	
Comorbidities	None	112 (74.2%)	54 (76.1%)	58 (72.5%)	0.760
	One	33 (21.9%)	15 (21.1%)	18 (22.5%)	
	Two	6 (4.0%)	2 (2.8%)	4 (5.0%)	

Abbreviations: EDSS—Expanded Disability Status Scale; SPMS—secondary-progressive multiple sclerosis; PPMS—primary-progressive multiple sclerosis; RRMS—relapsing-remitting multiple sclerosis and DMT—disease-modifying treatment.

**Table 2:** Spearman correlation of ICF categories and work capacity. *** *p* < 0.001.

ICF Code and Category Title	rs Work Capacity of Participants with MS	ICF Code and Category Title	rs Work Capacity of Participants with MS
**Body functions**			
b134 Sleep functions	−0.292 ***	b730 Muscle power functions	−0.681 ***
b164 Higher-level cognitive functions	−0.356 ***	b735 Muscle tone functions	−0.393 ***
b260 Proprioceptive function	−0.450 ***	b740 Muscle endurance functions	−0.538 ***
b455 Exercise tolerance functions	−0.418 ***	b750 Motor reflex functions	−0.451 ***
b5105 Swallowing	−0.250 ***	b760 Control of voluntary movement functions	−0.316 ***
b525 Defecation functions	−0.427 ***	b7650 Involuntary contractions of muscles	−0.295 ***
b620 Urination functions	−0.436 ***	b770 Gait pattern functions	−0.704 ***
**Activities and Participation**			
d155 Acquiring skills	−0.280 ***	d510 Washing oneself	−0.501 ***
d170 Writing	−0.353 ***	d520 Caring for body parts	−0.490 ***
d175 Solving problems	−0.335 ***	d620 Acquisition of goods and services	−0.536 ***
d220 Undertaking multiple tasks	−0.407 ***	d630 Preparing meals	−0.432 ***
d240 Handling stress and other psychological demands	−0.290 ***	d640 Doing housework	−0.538 ***
d410 Changing basic body position	−0.509 ***	d650 Caring for household objects	−0.443 ***
d415 Maintaining a body position	−0.574 ***	d720 Complex interpersonal interactions	−0.281 ***
d420 Transferring oneself	−0.542 ***	d750 Informal social relationships	−0.341 ***
d430 Lifting and carrying objects	−0.357 ***	d845 Acquiring, keeping and terminating a job	−0.379 ***
d440 Fine hand use	−0.407 ***	d850 Remunerative employment	−0.307 ***
d445 Hand and arm use	−0.445 ***	d860 Basic economic transactions	−0.314 ***
d450 Walking	−0.587 ***	d870 Economic self−sufficiency	−0.359 ***
d455 Moving around	−0.452 ***	d910 Community life	−0.288 ***
d460 Moving around in different locations	−0.657 ***	d920 Recreation and leisure	−0.422 ***
d470 Using transportation	−0561 ***		

**Table 3:** Results of final step of multiple regression analysis in predicting work capacity.

Variables in the Equation	B (SE)	Beta	t	*p*
b770 Gait pattern functions	−3.501 (0.771)	−0.307	−4.543	<0.001
b730 Muscle power functions	−4.299 (0.723)	−0.376	−5.946	<0.001
b134 Sleep functions	−2.138 (0.539)	−0.194	−3.967	<0.001
Age	−0.124 (0.047)	−0.134	−2.627	0.010
d845 Acquiring, keeping and terminating a job	−0.581 (0.252)	−0.119	−2.304	0.023
b620 Urination functions	−1.226 (0.609)	−0.109	−2.012	0.046

**Table 4:** Results of the final step of logistic regression analysis in predicting employment status.

Variables in the Equation	B (SE)	Wald (df = 1)	*p*	Exp (B)
b164 Higher-level cognitive functions	−1.926 (0.682)	7.977	0.005	0.146
d330 Speaking	4.556 (1.564)	8.483	0.004	95.239
d455 Moving around	−0.452 (0.186)	5.927	0.015	0.636
d510 Washing oneself	−3.178 (0.868)	13.401	<0.001	0.042
d630 Preparing meals	1.928 (0.865)	4.969	0.026	6.878
d870 Economic self-sufficiency	−2.966 (0.590)	25.254	<0.001	0.051
d910 Community life	1.327 (0.568)	5.447	0.020	3.768
Education	0.773 (0.246)	9.862	0.002	2.167

## Data Availability

Any request for data by qualified scientific and medical researchers for legitimate research purposes should be submitted by contacting the first author (daiva.valadkeviciene@mf.vu.lt).

## Supplementary Materials

The following supporting information can be downloaded at: https://www.mdpi.com/article/10.3390/jcm13144195/s1, Table S1: ICF categories referring to body functions, body structures, and activities and participation are reported as at least mildly impaired in more than 10% of participants and are ordered by frequency (n) (*n* = 151); Figure S1: Flow chart diagram of the study cohort selection process.
