# Supplementary material for: Can International Classification of Functioning, Disability and Health (ICF) Be Used for Prediction of Work Capacity and Employment Status in Multiple Sclerosis?
Source: J Clin Med. 2024 Jul 18;13(14):4195. doi: 10.3390/jcm13144195 (PMC11277909; doi:10.3390/jcm13144195)
Supplement: Supplementary file 1 [file jcm-13-04195-s001.zip › jcm-3073263-supplementary.pdf]

Supplemental Table S1. ICF categories referring to 'body functions', 'body structures', and 'activities and participation' are reported as at least mildly impaired in more than 10% of participants and are ordered by frequency (n) (N=151)

		total	1*	2*	3*	4*
**Body functions**	(%)	n	n	n	n	n
b455 Exercise tolerance functions	94.0%	142	76	58	5	3
b730 Muscle power functions	92.1%	139	66	57	12	4
b770 Gait pattern functions	90.7%	137	83	35	17	2
b760 Control of voluntary movement functions	88.7%	134	62	49	23	0
b620 Urination functions	81.5%	123	77	36	7	3
b750 Motor reflex functions	81.5%	123	52	39	31	1
b1300 Energy level	72.8%	118	98	16	4	0
b126 Temperament and personality functions	74.8%	113	93	18	2	0
b210 Seeing functions	74.2%	112	79	27	6	0
b740 Muscle endurance functions	73.5%	111	74	31	5	1
b144 Memory functions	70.2%	106	89	16	1	0
b5500 Body temperature	68.2%	103	83	19	0	1
b7650 Involuntary contractions of muscles	50.3%	98	76	19	3	0
b260 Proprioceptive function	64.2%	97	76	17	4	0
b280 Sensation of pain	64.2%	97	57	37	3	0
b140 Attention functions	57.0%	86	80	5	1	0
b134 Sleep functions	54.3%	82	53	20	9	0

b265 Touch function	54.3%	82	68	13	1	0
b525 Defecation functions	52.3%	79	59	14	3	3
b1301 Motivation	50.7%	76	69	5	2	0
b152 Emotional functions	46.4%	70	37	30	3	0
b330 Fluency and rhythm of speech functions	46.4%	70	62	8	0	0
b735 Muscle tone functions	44.0%	66	32	23	11	0
b780 Sensations related to muscles and movement functions	43.0%	65	55	9	1	0
b5105 Swallowing	40.4%	61	46	13	2	0
b7651 Tremor	37.1%	56	45	9	2	0
b164 Higher-level cognitive functions	35.1%	53	47	5	1	0
b270 Sensory functions related to temperature and other stimuli	23.8%	36	27	8	1	0
b114 Orientation functions	20.5%	31	28	1	2	0
b5104 Salivation	18.5%	28	26	2	0	0
b320 Articulation functions	11.9%	18	11	7	0	0
**Body Structures**		total	1*	2*	3*	4*
s110 Structure of brain	98.7%	149	1	28	109	11
s120 Spinal cord and related structures**	88.1%	59	16	23	19	1
**Activities & Participation**		total	1*	2*	3*	4*
d455 Moving around	94.7%	143	34	10	7	92
d450 Walking	84.1%	127	49	29	22	27
d850 Remunerative employment	70.9%	107	0	0	0	107
d415 Maintaining a body position	70.2%	106	65	22	9	10

d460 Moving around in different locations	68.9%	104	47	29	18	10
d920 Recreation and leisure	64.2%	97	87	5	1	4
d240 Handling stress and other psychological demands	60.3%	91	71	20	0	0
d465 Moving around using equipment***	92.8%	90	38	38	13	1
d845 Acquiring, keeping and terminating a job	53.6%	81	0	0	0	81
d870 Economic self-sufficiency	53.6%	81	68	8	3	2
d220 Undertaking multiple tasks	52.3%	79	64	10	3	2
d410 Changing basic body position	49.0%	74	43	24	4	3
d475 Driving****	58.9%	73	24	8	3	38
d445 Hand and arm use	43.7%	66	56	5	2	3
d620 Acquisition of goods and services	43.7%	66	34	19	5	8
d650 Caring for household objects	39.7%	60	39	11	4	6
d170 Writing	36.4%	55	45	7	3	0
d640 Doing housework	36.4%	55	34	13	2	6
d440 Fine hand use	35.8%	54	43	6	3	2
d420 Transferring oneself	34.4%	52	41	5	4	2
d770 Intimate relationships	33.1%	50	7	6	2	35
d910 Community life	32.5%	49	43	2	1	3
d470 Using transportation	27.8%	42	21	5	8	8
d760 Family relationships	27.8%	42	28	0	0	14
d430 Lifting and carrying objects	25.8%	39	30	7	2	0
d520 Caring for body parts	25.8%	39	29	4	4	2
d630 Preparing meals	23.2%	35	27	2	1	5

d510 Washing oneself	21.9%	33	21	5	6	1
d720 Complex interpersonal interactions	21.9%	33	31	1	0	1
d166 Reading	20.5%	31	22	6	2	1
d155 Acquiring skills	19.9%	30	25	4	1	0
d160 Focusing attention	17.2%	26	26	0	0	0
d175 Solving problems	14.6%	22	18	3	1	0
d860 Basic economic transactions	13.2%	20	16	0	2	2
d230 Carrying out daily routine	12.6%	19	17	1	1	0
d660 Assisting others	12.6%	19	18	0	0	1
d750 Informal social relationships	11.3%	17	15	0	1	1
d830 Higher education	10.6%	16	8	1	0	7
**Environmental Factors (distribution of barriers)**		total	-1*	-2*	-3*	-4*
e2250 Temperature	57.6%	87	82	5	0	0
e590 Labour and employment services, systems and policies	51.7%	78	0	0	0	78
e2251 Humidity	23.8%	36	33	3	0	0
e2253 Precipitation	21.9%	33	31	2	0	0
e330 People in positions of authority	15.2%	23	14	3	1	5
e115 Products and technology for personal use in daily living	13.2%	20	18	0	1	1
e165 Assets	12.6%	19	18	1	0	0
e310 Immediate family	10.6%	16	12	4	0	0

The table presents the absolute and relative frequencies (%) of identified categories for the total sample.

* 1 mild impairment/restriction, 2 moderate impairment/restriction, 3 severe impairment/restriction, 4 complete impairment/restriction

** only 67 patients had scores of s120;

*** only 97 patients had scores of d465;

**** only 124 patients had scores of d475.

Supplementary Figure S1. Flow chart diagram of the study cohort selection process.

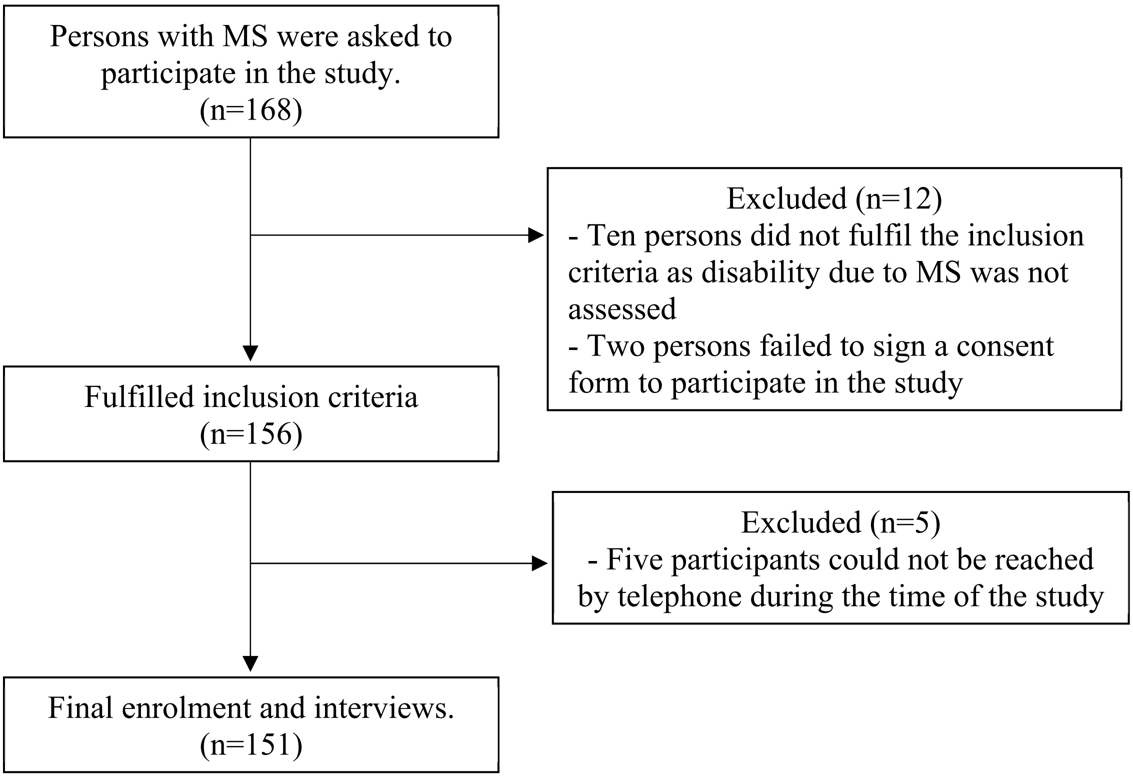

Abbreviation: MS – Multiple sclerosis.
